# Periodic Bleeding from a Cesarean Section Scar Fistula

**DOI:** 10.3390/diagnostics14212403

**Published:** 2024-10-29

**Authors:** Gilbert Georg Klamminger, Daniel-Christoph Wagner, Martin Beeres, Annette Hasenburg, Roxana Schwab

**Affiliations:** 1 Department of Obstetrics and Gynecology, University Medical Center of Johannes Gutenberg University Mainz, 55131 Mainz, Germany; 2 Institute of Pathology, University Medical Center of Johannes Gutenberg University Mainz, 55131 Mainz, Germany; 3 Institute for Diagnostic and Interventional Radiology, Goethe University Frankfurt, 60596 Frankfurt, Germany; 4 Department of Neuroradiology, University Hospital Giessen and Marburg, 35043 Marburg, Germany

**Keywords:** catamenial scar bleeding, cesarean section scar fistula, fistula

## Abstract

We present the case of a 36-year-old woman who presented to our clinic for a second opinion. After multiple previous surgeries, she presented with an abdominal wound infection, which was initially treated conservatively. In the further course, catamenial bleeding occurred as an unusual symptom, and the suspicion of a fistula formation was postulated. Subsequent surgical repair and consecutive histopathological diagnosis revealed evidence of a fistula with endometriosis genitalis externa and thus gave an explanation for this striking clinical case presentation.

A 36-year-old female patient (gravida IV, para IV) presented to our clinic 10 weeks postpartum following an elective repeat cesarean section. Due to a postpartum puerperal infection and a symptomatic ovarian cyst (cystadenoma), a re-laparotomy was performed 3 weeks after the cesarean section.

She presented with ongoing complications of wound healing and wound infection around the cesarean scar. Microbiological investigations had already confirmed the presence of a polymicrobial colonization (*Staphylococcus aureus*, *Streptococcus* spp., *Klebsiella* spp., *Corynebacteria*), and antibiotic therapy (clindamycin) had been initiated ex domo. During the clinical examination of the abdomen and the cesarean scar, a large amount of purulent exudate was observed, along with three dehiscent areas (up to 0.5 cm in diameter) within the scar. Consecutive blood testing revealed a C-reactive protein (CRP) level of 47 mg/L and an almost normal leukocyte count (10.8/nL). A previously extern-recommended indication for surgical wound management using vacuum-assisted closure (VAC) therapy was confirmed at our clinic; however, the patient refused this treatment.

Six months later, the patient returned to our clinic, reporting cyclic pain and catamenial bleeding from the area of the persistent wound dehiscence during menstruation. Up to this point, dysmenorrhea, dyspareunia, chronic pelvic pain or other endometriosis-related symptoms had not been reported, nor had any preceding abdominal surgery revealed signs of endometriosis. Our clinical examination raised suspicion of a fistulous tract between the abdominal wall and the anterior wall of the uterus in the midline of the cesarean scar. This suspicion was further supported by magnetic resonance imaging (MRI) of the abdomen, which suggested a distinct contact area between the uterus and the abdominal wall (Figure 1), along with impaired wound healing and fascial dehiscence in the context of the previous cesarean section.

The patient was consented to a re-laparotomy with excision of the fistulous tract, adhesiolysis, and reconstruction of both the uterus and the abdominal fascia. Subsequent histological examination confirmed the presence of ectopic endometrial glandular structures and surrounding cytogenic stroma with signs of chronic inflammation within the fistulous tract (Figure 2) [1], consistent with the phenomenon of abdominal wall endometriosis post-cesarean section [2].

In contrast to the occasionally reported post-cesarean scar endometriosis which typically presents as an abdominal mass/lesion with periodically pain [3,4,5], our patient reported cyclic recurrent bleeding and fistulous tract formation. From an etiopathological perspective, several factors—such as patient-related risk factors (obesity, deficient nutritional status) as well as pathogenic microbial colonization of the wound area and the surgical technique/trauma itself (insufficient suturing, extensive tissue trauma, change in uterine position or corporal incision and consecutive cesarean scar defects)—may have contributed to the presented affection [6,7,8]. On the one hand our case demonstrates the urgency for clinical reasoning in identifying patients at risk for cesarean section and following postoperative complications, allowing timely and well-planned pre-surgical preparations [9,10]. On the other hand, it emphasizes the necessity of good surgical practice in gynecological abdominal surgery/cesarean section delivery (proper anatomical layering, appropriate incision and closure techniques, adequate use of surgical instruments, correct employment of cauterization, vaginal preparation, antibiotics prophylaxis, as well as suitable suture materials), avoiding not only short-term complications but also long-term impairments [11,12,13].

In our presented case, a postoperative follow-up examination revealed a well-healed wound/scar formation with intact abdominal wall structure. No evidence of recurrent adhesions was found by transvaginal and abdominal ultrasound examination.

## Figures and Tables

**Figure 1:**
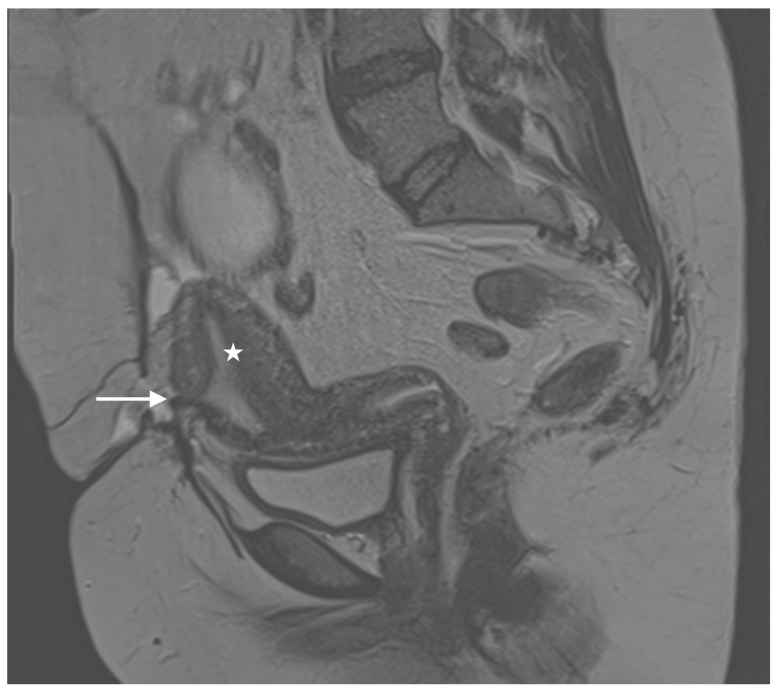
Diagnostic imaging (MRI, T2) shows a scar region (arrow) within the ventral corpus uteri. The corpus uteri (asterisk) is displaced ventrally towards the abdominal wall. In alignment with the clinical symptoms, the findings are highly suspicious for fistula formation.

**Figure 2:**
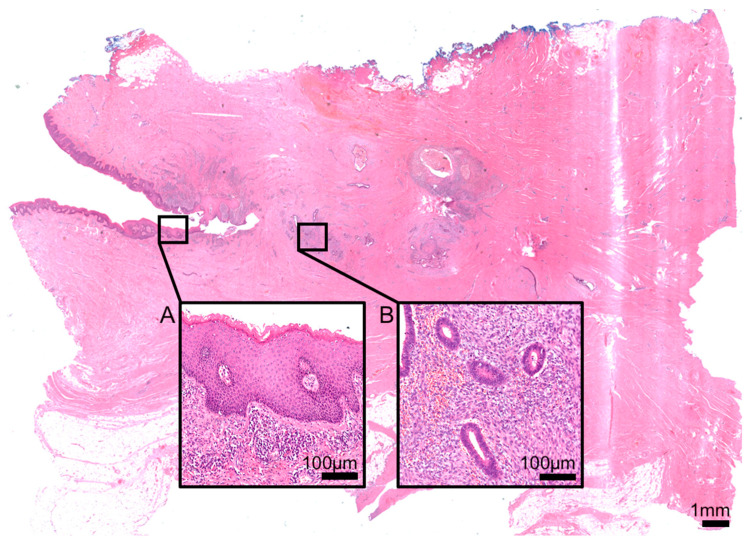
Histomorphologically, next to signs of chronic inflammation as well as the umbilical surface (A), ectopic endometrial mucosa was detected (B).

## Data Availability

The data presented in this study are available on request from the corresponding author. The data are not publicly available due to individual solutions.

## References

[1] Istrate-Ofiţeru A.-M., Mogoantă C.A., Zorilă G.-L., Roşu G.-C., Drăguşin R.C., Berbecaru E.-I.-A., Zorilă M.V., Comănescu C.M., Mogoantă S.- (2024). Ștefăniță; Vaduva, C.-C.; et al. Clinical Characteristics and Local Histopathological Modulators of Endometriosis and Its Progression. Int. J. Mol. Sci..

[2] WHO Classification of Tumours Editorial Board (2020). Female Genital Tumours. WHO Classification of Tumours.

[3] Horton J.D., Dezee K.J., Ahnfeldt E.P., Wagner M. (2008). Abdominal Wall Endometriosis: A Surgeon’s Perspective and Review of 445 Cases. Am. J. Surg..

[4] Arkoudis N.-A., Moschovaki-Zeiger O., Prountzos S., Spiliopoulos S., Kelekis N. (2023). Caesarean-Section Scar Endometriosis (CSSE): Clinical and Imaging Fundamentals of an Underestimated Entity. Clin. Radiol..

[5] Zhang P., Sun Y., Zhang C., Yang Y., Zhang L., Wang N., Xu H. (2019). Cesarean Scar Endometriosis: Presentation of 198 Cases and Literature Review. BMC Womens Health.

[6] Pan H., Gu A., Yang Y., Chen Z., Liang F. (2022). Postpartum Changes in Uterine Position and Occurrence of Cesarean Scar Defects: A Retrospective Observational Study. Clin. Exp. Obstet. Gynecol..

[7] Donnez O. (2023). Cesarean Scar Disorder: Management and Repair. Best Pract. Res. Clin. Obstet. Gynaecol..

[8] Antila-Långsjö R.M., Mäenpää J.U., Huhtala H.S., Tomás E.I., Staff S.M. (2018). Cesarean Scar Defect: A Prospective Study on Risk Factors. Am. J. Obstet. Gynecol..

[9] Frappaolo A.M., Logue T.C., Goffman D., Nathan L.M., Sheen J.-J., Andrikopoulou M., Wen T., D’Alton M.E., Friedman A.M. (2023). Cesarean Delivery Trends Among Patients at Low Risk for Cesarean Delivery in the US, 2000–2019. JAMA Netw. Open.

[10] Cavoretto P.I., Candiani M., Farina A. (2023). Cesarean Delivery Uptake Trends Associated With Patient Features and Threshold for Labor Anomalies. JAMA Netw. Open.

[11] Chintamani (2018). Ten Commandments of Safe and Optimum Abdominal Wall Closure. Indian J. Surg..

[12] Haas D.M., Morgan S., Contreras K., Enders S. (2018). Vaginal Preparation with Antiseptic Solution before Cesarean Section for Preventing Postoperative Infections. Cochrane Database Syst. Rev..

[13] Smaill F.M., Grivell R.M. (2014). Antibiotic Prophylaxis versus No Prophylaxis for Preventing Infection after Cesarean Section. Cochrane Database Syst. Rev..

